# A predictive model of thyroid malignancy using clinical, biochemical and sonographic parameters for patients in a multi-center setting

**DOI:** 10.1186/s12902-018-0241-7

**Published:** 2018-03-07

**Authors:** Jia Liu, Dongmei Zheng, Qiang Li, Xulei Tang, Zuojie Luo, Zhongshang Yuan, Ling Gao, Jiajun Zhao

**Affiliations:** 1 Department of Endocrinology, Shandong Provincial Hospital Affiliated to Shandong University, Jinan, Shandong 250021 China; 2 Shandong Clinical Medical Center of Endocrinology and Metabolism, Jinan, Shandong 250021 China; 3 Institute of Endocrinology and Metabolism, Shandong Academy of Clinical Medicine, Jinan, Shandong 250021 China; 4 Department of Endocrinology and Metabolism, the Second Affiliated Hospital of Harbin Medical University, Harbin, Heilongjiang 150086 China; 5 Department of Endocrinology, the First Hospital of Lanzhou University, Lanzhou, Gansu 730000 China; 6 Department of Endocrinology, the First Affiliated Hospital of Guangxi University, Nanning, Guangxi 530021 China; 7 Department of Biostatistics, School of Public Health, Shandong University, Jinan, Shandong 250021 China; 8 Department of Endocrinology and Metabolism, Shandong Provincial Hospital Affiliated to Shandong University, Jingwu Road 324, Jinan, Shandong 250021 China

**Keywords:** Thyroid nodules, Malignancy, Predictive model

## Abstract

**Background:**

Thyroid nodules are highly prevalent, but a robust, feasible method for malignancy differentiation has not yet been well documented. This study aimed to establish a practical model for thyroid nodule discrimination.

**Methods:**

Records for 2984 patients who underwent thyroidectomy were analyzed. Clinical, laboratory, and US variables were assessed retrospectively. Multivariate logistic regression analysis was performed and a mathematical model was established for malignancy prediction.

**Results:**

The results showed that the malignant group was younger and had smaller nodules than the benign group (43.5 ± 11.6 vs. 48.5 ± 11.5 y, *p* < 0.001; 1.96 ± 1.16 vs. 2.75 ± 1.70 cm, *p* < 0.001, respectively). The serum thyrotropin (TSH) level (median = 1.63 mIU/L, IQR (0.89–2.66) vs. 1.19 (0.59–2.10), *p* < 0.001) was higher in the malignant group than in the benign group. Patients with malignancies tested positive for anti-thyroglobulin antibody (TGAb) and anti-thyroid peroxidase antibody (TPOAb) more frequently than those with benign nodules (TGAb, 30.3% vs. 15.0%, *p* < 0.001; TPOAb, 25.6% vs. 18.0%, *p* = 0.028). The prevalence of ultrasound (US) features (irregular shape, ill-defined margin, solid structure, hypoechogenicity, microcalcifications, macrocalcifications and central intranodular flow) was significantly higher in the malignant group. Multivariate logistic regression analysis confirmed that age (OR = 0.963, 95% CI = 0.934–0.993, *p* = 0.017), TGAb (OR = 4.435, 95% CI = 1.902–10.345, *p* = 0.001), hypoechogenicity (OR = 2.830, 95% CI = 1.113–7.195, *p* = 0.029), microcalcifications (OR = 4.624, 95% CI = 2.008–10.646, *p* < 0.001), and central intranodular flow (OR = 2.155, 95% CI = 1.011–4.594, *p* < 0.05) were independent predictors of thyroid malignancy. A predictive model including four variables (age, TGAb, hypoechogenicity and microcalcification) showed an optimal discriminatory accuracy (area under the curve, AUC) of 0.808 (95% CI = 0.761–0.855). The best cut-off value for prediction was 0.52, achieving sensitivity and specificity of 84.6% and 76.3%, respectively.

**Conclusion:**

A predictive model of malignancy that combines clinical, laboratory and sonographic characteristics would aid clinicians in avoiding unnecessary procedures and making better clinical decisions.

**Electronic supplementary material:**

The online version of this article (10.1186/s12902-018-0241-7) contains supplementary material, which is available to authorized users.

## Background

Thyroid nodules are highly prevalent in the general adult population, with a detection rate of 19–67% during routine ultrasound examinations [1]. An epidemiological study showed that approximately 5–15% of these nodules are malignant [2]. Despite the high incidence of thyroid malignancy, most patients referred for suspected nodules have benign conditions. The overestimation of malignancy leads to the performance of unnecessary procedures and causes a burden for both society and patients. Therefore, distinguishing thyroid nodules preoperatively is required.

To date, the Thyroid Imaging Reporting and Data System (TIRADS) and American Thyroid Association guidelines are considered as the main criteria for determining malignancy and are generally followed by radiologists in practice [3]. However, these categorization systems were established based on fine needle aspiration (FNA) cytology results that included data from nodules > 1 cm. In addition, a few reports have presented serum thyrotropin (TSH) and positive thyroid autoantibodies as possible predictors of thyroid malignancy [4, 5]. However, these guidelines or studies either used FNA cytology results for their final diagnoses, which are less reliable than those confirmed via surgical inspection, or they included a relatively small number of patients. Additionally, most studies to date have focused on single risk factors, clinical, biochemical or radiological, and only a few studies have analyzed these risk factors in combination. A robust predictive model involving easily accessible clinical, laboratory and radiological risk factors may serve as a pragmatic aid in making decisions regarding malignancy differentiation.

In the present study, we reviewed a large cohort of 2984 patients in China who underwent thyroid surgery and had final pathological data available. The purpose of our study was to verify the independent risk factors of clinical, laboratory and ultrasonographic (US) features in patients with thyroid carcinomas and to establish a predictive model for determining malignancy that can be used by clinical practitioners.

## Methods

### Patients

We retrospectively studied the data from 3145 consecutive patients who mostly received routine neck ultrasound detections and underwent total or partial thyroid surgery between 2006 and 2009 at four tertiary hospitals in China. Patients with a previous thyroid surgery or radiation ablation and patients who were taking thyroxine or antithyroid drugs were not included. Patients with medullary thyroid cancer, anaplastic cancer or lymphoma were considered TSH-nonresponsive and were excluded. After the exclusions, 2984 patients were included in the analysis. Their clinical, laboratory, and US variables were assessed retrospectively. This study had institutional review board approval.

### US imaging analysis

US examinations of the four tertiary hospitals were performed using US scanner GE LOGIQ9 (USA) equipped with a 5–12-MHz linear transducer for morphological examinations and a 4.7-MHz transducer for color Doppler evaluations. The examinations were conducted and recorded by two skilled sonographers from respective hospitals according to a standard procedure and interobservers reached agreement on the results of each US findings. The following US parameters of the nodules were recorded: (1) number of nodules, (2) nodule size, (3) echoic texture, (4) echogenicity, (5) shape, (6) margin, (7) calcification (microcalcification, macrocalcification, or egg-shell calcification) and (8) intranodular central flow.

### Laboratory variables

The levels of serum TSH, free triiodothyronine (FT3) and free thyroxine (FT4) were determined using chemiluminescence analyzer Roche Cobas E601 (Switzerland) and the matched kit. These values ranged from 0.35 to 5.5 UI/ml for TSH, from 11.5 to 22.7 pmol/l for FT4 and from 3.5 to 6.5 pmol/l for FT3. If the other laboratories had different normal ranges, the values were adjusted to reflect the same normal range. Anti-thyroid peroxidase antibody (TPOAb, reference value < 60 μIU/ml) and anti-thyroglobulin antibody (TGAb, reference value < 60 IU/ml) levels were measured using immunometric assays. Thyroid antibody levels higher than the upper range were considered positive.

### Pathology

FNA cytology was not generally performed and considered as a routine pre-operative assessment when the study was conducted. Postoperative histopathologic evaluations were performed by pathologists experienced in thyroid pathology. The histopathologic results of the patients operated on were grouped as either malignant or benign.

### Statistical analysis

Descriptive statistics are presented as the means ± standard deviations for continuous variables and as the number of patients and percentages for categorical variables. Differences between independent groups for continuous variables were evaluated using a Student’s t-test or a Mann–Whitney U-test, where applicable. Categorical data were analyzed using Pearson’s chi-square test. Univariate and multivariate logistic regression analyses were performed to evaluate the association between malignancy and risk factors. Appealing receiver operating characteristic (ROC) curve analyses were performed to examine the predictive power of combinations of clinical, laboratory and sonographic features. The areas under the curves (AUCs) were derived from ROC curves. The Youden index was used to define the optimal cut-off value [6]. All statistical analyses were performed using SPSS version 17.0 (SPSS, Inc., Chicago, IL). Differences between AUCs were detected using Delong’s test [7]. A *p*-value of < 0.05 was considered statistically significant.

## Results

### Clinical characteristics

This study cohort consisted of 541 men and 2443 women. Overall, 2460 patients were diagnosed with pathologically benign nodules, and 524 patients were diagnosed with malignant nodules. The malignancy rate in our study was 17.6%. Most of the nodules were detected incidentally in routine body check-up and totally 10.5% of the patients present clinical systems such as hoarsennes, swallowing difficulty, thyroid enlargement, with the duration of symptoms varying from 7 days to 26 years. As shown in Table 1, there was no difference in the sex ratios between the patients with benign and malignant nodules. Patients with malignant nodules were younger than those without malignant nodules (43.5 ± 11.6 years vs. 48.5 ± 11.5 years, *p* < 0.001) (Table 1).

**Table 1:** Clinical characteristics of 2984 subjects with thyroid nodules

	Benign (*n* = 2460)	Malignant (*n* = 524)	*P* value
Gender			
Male,%	17.7%	20.0%	0.212
Age, y, mean(SD)	48.5(11.5)	43.5(11.6)	< 0.001
Nodule size, cm, mean(SD)	2.75(1.70)	1.96(1.16)	< 0.001
Solitary nodule, %	25.1%	29.0%	0.109

Continuous variables were compared using Student’s tests or Mann-Whitney U tests, and categorical variables, using X^2^tests. *P* < 0.05 was considered significant. Nodule size was derived from ultrasound detection

The mean maximal diameter of malignant nodules was significantly smaller than that of benign nodules (1.96 ± 1.16 cm vs. 2.75 ± 1.70 cm, *p* < 0.001). The prevalence of solitary nodules in malignant cases was not different from that in benign cases (29.0% vs. 25.1%, *p* = 0.109).

### Laboratory values

**Fig. 1:**
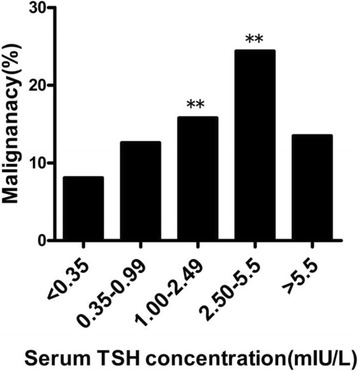
Prevalence of malignancy in relation to the serum TSH concentration, indicating an increased prevalence in patients with higher TSH levels. ^**^P < 0.05, compared with patients with TSH levels less than 0.35 mIU/L

As shown in Table 2, there were no significant differences in FT3 and FT4 values between the two groups. The level of TSH (median 1.63 mIU/L, IQR (0.89–2.66) vs. 1.19 (0.59–2.10), p < 0.001] in the malignant group was higher than in the benign group. Subsequently, based on the cutoff values predetermined in population studies, TSH levels were divided into quintiles, including below normal (< 0.35 mIU/L), above normal (> 5.5 mIU/L), and within normal, with the latter divided into tertiles of similar size (0.35–0.99 mIU/L, 1.0–2.49 mIU/L, and 2.5–5.49 mIU/L). The prevalence of malignancy was 9.8% when TSH levels were less than 0.35 mIU/L, compared with 13.2% when TSH levels were 5.5 mIU/L or greater (*p* = 0.17). In the normal range, a high rate of malignancy was observed in patients with higher TSH levels. The prevalence of malignancy was 15.8% when TSH levels were between 1.0 and 2.49 mIU/L and 24.4% when TSH levels were between 2.50 and 5.49 mIU/L, compared with 12.6% when TSH levels were between 0.35 and 0.99 mIU/L (*p* = 0.09 and *p* < 0.001, respectively) (Fig. 1).

**Table 2:** Laboratory variables of subjects with thyroid nodules

	Benign	Malignant	P value
FT3, pmol/L, median (IQR)	4.43 (3.91–5.07)	4.44 (3.96–5.00)	0.809
FT4, pmol/L, median (IQR)	14.97 (12.84–17.42)	15.76 (13.6–18.09)	0.064
TSH, mIU/ml, median (IQR)	1.19 (0.59–2.10)	1.63 (0.89–2.66)	< 0.001
TGAb, %	15.0%	30.3%	< 0.001
TPOAb, %	18.0%	25.6%	0.028

Continuous variables were compared using Mann-Whitney U tests, and categorical variables, using X^2^ tests. *P* < 0.05 was considered significant

*Abbreviations*: *FT3* free triiodothyronine, *FT4* free thyroxine, *TSH* thyrotropin, *TGAb* anti-thyroglobulin antibody, *TPOAb* anti-thyroid peroxidase antibody

Patients with malignant nodules had positive TGAb and TPOAb results more frequently than did patients with benign nodules (for TGAb, 30.3% vs. 15.0%, *p* < 0.001; for TPOAb, 25.6% vs. 18.0%, *p* = 0.028).

### Sonographic features

The prevalences of an irregular shape (42.7% vs. 10.7%, *p* < 0.001), an ill-defined margin (38.7% vs. 9.7%, *p* < 0.001), a solid structure (75.8% vs. 41.3% *p* < 0.001), hypoechogenicity (68.5% vs. 27.1%, *p* < 0.01), microcalcification (48.5% vs. 13%, *p* < 0.001), macrocalcification (18.5% vs. 12.5%, *p* = 0.001), and an intranodular central flow (60.3% vs. 47.1%, *p* < 0.001) were significantly higher in malignant nodules than in benign nodules (Table 3). There were no differences between the benign and malignant groups for egg-shell calcifications (*p* > 0.05).

**Table 3:** Sonographic features of subjects with thyroid nodules

	Benign	Malignant	P value
Irregular shape	10.7%	42.7%	< 0.001
Ill-defined margin	9.7%	38.7%	< 0.001
Solid structure	41.3%	75.8%	< 0.001
Hypoechogenicity	27.1%	68.5%	< 0.01
Microcalcification	13.0%	48.5%	< 0.001
Macrocalcification	12.5%	18.5%	0.001
Egg-shell calcification	1.6%	1.7%	0.797
Central flow	47.1%	60.3%	< 0.001

Categorical variables were compared using X^2^ tests. *P* < 0.05 was considered significant

### Clinical, biochemical and sonographic characteristics of microcarcinoma

Of 524 malignant nodules, 104 nodules ≤1 cm in diameter were defined as microcarcinomas. Since microcarcinoma is considered “more silent”, we analyzed clinical, biochemical and sonographic parameters separately. As shown in the Additional file 1: Table S1, we found age, positive TGAb result, hypoechogenicity, microcalcification and intranodular central flow were also associated with increased risk for malignancy in the nodules less than 1 cm in diameter.

### The associations between risk factors and the presence of malignant nodules

We further explored the correlation of clinical characteristics, laboratory values and US features with the risk for malignant nodules via univariate analysis, which gave results consistent with those from the prevalence analysis (data not shown). Multivariate analysis confirmed that age had a significant negative correlation with an increased risk of thyroid malignancy (OR 0.963, 95% CI 0.934–0.993, *p* = 0.017) (Table 4). Additionally, a positive TGAb result, hypoechogenicity, microcalcification and intranodular central flow were independently associated with increased risks for malignant nodules (TGAb OR 4.435, 95% CI 1.902–10.345, *p* = 0.001; hypoechogenicity OR 2.830, 95% CI 1.113–7.195, *p* = 0.029; microcalcification OR 4.624, 95% CI 2.008–10.646, *p* < 0.001; central flow OR 2.155, 95% CI 1.011–4.594, *p* < 0.05, respectively).

**Table 4:** Multivariate logistic regression of risk factors for the presence of thyroid malignancy

	B	SE	OR	95%CI of OR	P value
Age	−0.038	0.016	0.963	0.934–0.993	0.017
Nodule size	−0.262	0.153	0.770	0.571–1.038	0.086
TSH	0.024	0.056	1.025	0.918–1.143	0.664
TGAb	1.490	0.432	4.435	1.902–10.345	0.001
TPOAb	−0.104	0.489	0.901	0.346–2.350	0.832
Irregular shape	1.089	0.579	2.972	0.955–9.245	0.06
Ill-defined margin	0.099	0.626	1.104	0.324–3.767	0.874
Solid structure	−0.251	0.453	0.778	0.320–1.891	0.580
Hypoechogenicity	1.040	0.476	2.830	1.113–7.195	0.029
Microcalcification	1.531	0.426	4.624	2.008–10.646	< 0.001
Macrocalcification	0.961	0.514	2.614	0.955–7.154	0.061
Central flow	0.768	0.386	2.155	1.011–4.594	0.047

Data are coefficients (B), corresponding SE, OR, 95% CI, and measure of significance (P value)

*Abbreviations*: *CI* confidence interval, *OR* odds ratio

### The performance of independent risk factors—A mathematical model to predict malignancy

**Fig. 2:**
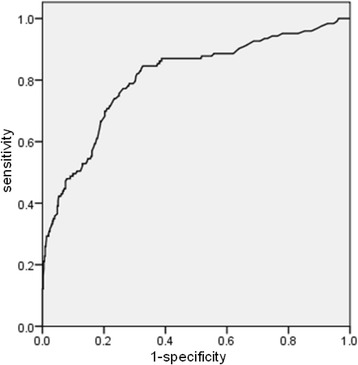
ROC curve for cancer prediction with a discrimination accuracy (AUC) of 0.808, 95%CI 0.761–0.855

To evaluate the predictive power of combinations of clinical characteristics, laboratory values and US features and to establish a mathematical model to calculate the risk for malignancy, a series of ROC curve analyses were performed, and AUCs were calculated. When the factors age, TGAb, hypoechogenicity and microcalcification were combined, the optimal AUC had a favorable value of 0.808 (0.761–0.855), indicating a diagnostic accuracy of 80.8% (Fig. 2). By combining these four independent risk factors of malignancy, we established the following formula for a predictive model:

p = (EXP(− 0.963–0.4*age + 1.108*TGAb+ 1.441*microcalcification+ 1.722*hypoechogenicity)/(1 + EXP(− 0.963–0.4*age + 1.108*TGAb+ 1.441*microcalcification+ 1.722*hypoechogenicity)).

The best cut-off value was calculated as 0.52, with a sensitivity of 84.6% and a specificity of 76.3%.

## Discussion

In this study, we verified risk factors associated with thyroid malignancy after comprehensively evaluating clinical, laboratory and sonographic variables in a population of 2984 patients who underwent thyroidectomy. Subsequently, we developed a mathematical model for cancer prediction, thereby providing a practical tool for clinicians to distinguish thyroid nodules preoperatively.

In agreement with previous studies, we identified that decreased age was one of the independent risk factors for thyroid cancer [8]. Malignant nodules were smaller than benign nodules (1.96 ± 1.16 cm vs. 2.75 ± 1.70 cm, *p* < 0.001). However, our multivariate logistic analysis did not confirm a predictive role of nodule size. This difference indicates that smaller nodules may not have a higher risk of malignancy because patients with larger nodules often have an increased likelihood of surgery for benign reasons, such as compressive symptoms, whereas patients with smaller nodules without any suspicious sonographic findings often select a conservative follow-up.

Higher TSH values, even within normal ranges, have been associated with a higher prevalence of thyroid malignancy in some studies [4, 5, 9, 10]. The results of our study are in agreement with those of previous studies, except for when TSH levels were higher than 5.5 mIU/l, which was not associated with a further increase in the prevalence of malignancy. This difference may be due to selection bias because we excluded patients who were taking thyroxine drugs; therefore, the number of patients with TSH levels > 5.5 mIU/L would have been quite small. However, in our study TSH lost its diagnostic value after being included in the multivariate logistic regression analysis, probably due to its weak role in predicting malignancy, which could be masked by including other co-effectors. Elevated TGAb, but not TPOAb, levels were a significant predictor of thyroid cancer, which is consistent with the findings of other reports [11–14]. Consistently, our study confirmed that the prevalence of lymphocytic thyroiditis was more frequent in malignant nodules (Additional file 2: Table S2). Additionally, our data also confirmed that patients with thyroiditis had positive TGAb more frequently than patients without thyroiditis (63.9% vs. 13.0%, *p* < 0.001).

Numerous studies have investigated the role of US findings in the diagnosis of malignant nodules [1, 15–17]. These studies state that hypoechogenicity, microcalcification, thyroid nodules with irregular margins, and intranodular vascularity are important features in determining the risk of malignancy. However, Cappelli et al. showed that an ill-defined margin was a nonspecific finding that could be seen for both benign and malignant nodules [18]. Consistent with these previous findings, we confirmed that microcalcifications, hypoechogenicity and intranodular central flow were associated with increased risks of malignancy. Our study did not find an association between egg-shell calcification and malignancy. Peripheral-rim or eggshell calcification has generally been considered to be an indicator of a benign nodule. However, a recently published study of thyroid nodules with eggshell calcifications reported that the findings of a peripheral halo and disruption of eggshell calcifications may be useful predictors of malignancy [19, 20]. Further studies are needed to confirm this observation.

Previously, some researchers have reported several systems for maligncy assessment [21–25]. Stojadinovic et al. established a model based on the performance of electrical impedance scanning (EIS) EIS, which was not routinely scheduled in clinics [21]. Zahir et al. showed a complicated two-step predictive model which was less accesible for clinicans [22]. Koike et al. included US features alone for differentiating non-follicular neoplasms > 5 mm [23]. Maia et al. evaluated malignancy risk based on patients from a single center [24]. Banks et al. analyzed 639 patients established a diagnostic model using the variables age, nodule size and FNA cytology [25]. Different from previous reports, in this study we enrolled 2984 patients from multiple tertiary medical centers, which greatly strengthens the evidence for diagnostic evaluations. Additionally, our mathematical model is derived from a combination of easily accessible clinical, biochemical and sonographic predictors, which improves the feasibility and practical appeal, thereby helping clinicians with decision making and reducing unnecessary invasions.

In addition, we analyzed predictive variables based on postoperative pathological inspections instead of FNA cytology examinations. Although FNA is considered to be an accurate and cost-effective method for evaluating thyroid nodules with a high diagnostic sensitivity and specificity [26], there are some limitations to diagnostic FNAs. First, FNA is recommended for nodules > 1 cm at their greatest dimension with a highly or intermediately suspicious sonographic pattern and for nodules > 1.5 cm at their greatest dimension with a minimally suspicious sonographic pattern [3]. Nodules smaller than 1 cm are difficult to distinguish via FNA cytology. Second, the performance of FNA is largely affected by the experience of radiologists, and the quality of the FNA procedure may affect the results. Reflecting these limitations, a number of previous studies have analyzed risk stratification based on FNA diagnoses [4, 26, 27] and have shown that it is less reliable than postoperative pathological examinations, which were used in our study.

However, there are some limitations to this study. The US feature of a node being taller than it is wide is considered to be a reliable indicator for thyroid malignancy. Unfortunately, these data were not available for the majority of the patients; therefore, this parameter was not included in the analysis. An algorithm including this US feature might improve the diagnostic accuracy of the predictive model in our study. Although less convincing than operative confirmations, FNA cytology is a relatively effective and robust method for identifying malignancies. Unfortunately, due to limitations relating to the skill with which FNAs are performed and a lack of compliance by patients, FNAs were not routinely performed in suspicious thyroid nodules in this study. Lastly, our study is retrospective, and prospective studies in a larger patient population are required to define and verify this model of risk prediction to improve clinical management.

## Conclusion

In summary, we analyzed 2984 patients who underwent thyroidectomy from multiple tertiary medical centers and established a practical model for predicting malignancies using a combination of simple and accessible clinical, biochemical and sonographic predictors. Prospective studies are required to validate this predictive model in a larger population.

## Additional files

Additional file 1: **Table S1.** Clinical, biochemical and sonographic parameters of thyroid nodules ≤1 cm. (DOCX 17 kb)

Additional file 2: **Table S2.** Distribution of thyroid malignancy in patients with and without lymphocytic thyroiditis. (DOCX 15 kb)

## Abbreviations

AUC: Area under curve
FNA: Fine needle aspiration
FT3: Free triiodothyronine
FT4: Free thyroxine
TGAb: Anti-thyroglobulin antibody
TPOAb: Anti-thyroid peroxidase antibody
TSH: Serum thyrotropin
US: Ultrasound

## References

[1] Frates MC, Benson CB, Charboneau JW, Cibas ES, Clark OH (2005). Management of thyroid nodules detected at US: Society of Radiologists in ultrasound consensus conference statement. Radiology.

[2] Frates MC, Benson CB, Doubilet PM, Kunreuther E, Contreras M (2006). Prevalence and distribution of carcinoma in patients with solitary and multiple thyroid nodules on sonography. J Clin Endocrinol Metab.

[3] Haugen BR, Alexander EK, Bible KC, Doherty GM, Mandel SJ (2016). 2015 American Thyroid Association management guidelines for adult patients with thyroid nodules and differentiated thyroid cancer: the American Thyroid Association guidelines task force on thyroid nodules and differentiated thyroid cancer. Thyroid.

[4] Boelaert K, Horacek J, Holder RL, Watkinson JC, Sheppard MC (2006). Serum thyrotropin concentration as a novel predictor of malignancy in thyroid nodules investigated by fine-needle aspiration. J Clin Endocrinol Metab.

[5] Polyzos SA, Kita M, Efstathiadou Z, Poulakos P, Slavakis A (2008). Serum thyrotropin concentration as a biochemical predictor of thyroid malignancy in patients presenting with thyroid nodules. J Cancer Res Clin Oncol.

[6] Youden WJ (1950). Index for rating diagnostic tests. Cancer.

[7] DeLong ER, DeLong DM, Clarke-Pearson DL (1988). Comparing the areas under two or more correlated receiver operating characteristic curves: a nonparametric approach. Biometrics.

[8] Baier ND, Hahn PF, Gervais DA, Samir A, Halpern EF (2009). Fine-needle aspiration biopsy of thyroid nodules: experience in a cohort of 944 patients. AJR Am J Roentgenol.

[9] Haymart MR, Repplinger DJ, Leverson GE, Elson DF, Sippel RS (2008). Higher serum thyroid stimulating hormone level in thyroid nodule patients is associated with greater risks of differentiated thyroid cancer and advanced tumor stage. J Clin Endocrinol Metab.

[10] Jung KW, Park S, Kong HJ, Won YJ, Boo YK (2010). Cancer statistics in Korea: incidence, mortality and survival in 2006-2007. J Korean Med Sci.

[11] Kim ES, Lim DJ, Baek KH, Lee JM, Kim MK (2010). Thyroglobulin antibody is associated with increased cancer risk in thyroid nodules. Thyroid.

[12] Chiovato L, Latrofa F, Braverman LE, Pacini F, Capezzone M (2003). Disappearance of humoral thyroid autoimmunity after complete removal of thyroid antigens. Ann Intern Med.

[13] Chung JK, Park YJ, Kim TY, So Y, Kim SK (2002). Clinical significance of elevated level of serum antithyroglobulin antibody in patients with differentiated thyroid cancer after thyroid ablation. Clin Endocrinol.

[14] Sands NB, Karls S, Rivera J, Tamilia M, Hier MP (2010). Preoperative serum thyroglobulin as an adjunct to fine-needle aspiration in predicting well-differentiated thyroid cancer. J Otolaryngol Head Neck Surg.

[15] Papini E, Guglielmi R, Bianchini A, Crescenzi A, Taccogna S (2002). Risk of malignancy in nonpalpable thyroid nodules: predictive value of ultrasound and color-Doppler features. J Clin Endocrinol Metab.

[16] Kim EK, Park CS, Chung WY, Oh KK, Kim DI (2002). New sonographic criteria for recommending fine-needle aspiration biopsy of nonpalpable solid nodules of the thyroid. AJR Am J Roentgenol.

[17] Moon WJ, Jung SL, Lee JH, Na DG, Baek JH (2008). Benign and malignant thyroid nodules: US differentiation--multicenter retrospective study. Radiology.

[18] Cappelli C, Castellano M, Pirola I, Cumetti D, Agosti B (2007). The predictive value of ultrasound findings in the management of thyroid nodules. QJM.

[19] Kim BM, Kim MJ, Kim EK, Kwak JY, Hong SW (2008). Sonographic differentiation of thyroid nodules with eggshell calcifications. J Ultrasound Med.

[20] Park M, Shin JH, Han BK, Ko EY, Hwang HS (2009). Sonography of thyroid nodules with peripheral calcifications. J Clin Ultrasound.

[21] Stojadinovic A, Peoples GE, Libutti SK, Henry LR, Eberhardt J (2009). Development of a clinical decision model for thyroid nodules. BMC Surg.

[22] Taghipour Zahir S, Binesh F, Mirouliaei M, Khajeh E, Noshad S (2013). Malignancy risk assessment in patients with thyroid nodules using classification and regression trees. J Thyroid Res.

[23] Koike E, Noguchi S, Yamashita H, Murakami T, Ohshima A (2001). Ultrasonographic characteristics of thyroid nodules: prediction of malignancy. Arch Surg.

[24] Maia FF, Matos PS, Silva BP, Pallone AT, Pavin EJ (2011). Role of ultrasound, clinical and scintigraphyc parameters to predict malignancy in thyroid nodule. Head Neck Oncol.

[25] Banks ND, Kowalski J, Tsai HL, Somervell H, Tufano R (2008). A diagnostic predictor model for indeterminate or suspicious thyroid FNA samples. Thyroid.

[26] Cooper DS, Doherty GM, Haugen BR, American Thyroid Association Guidelines Taskforce on Thyroid N, Differentiated Thyroid C (2009). Revised American Thyroid Association management guidelines for patients with thyroid nodules and differentiated thyroid cancer. Thyroid.

[27] Chang SH, Joo M, Kim H (2006). Fine needle aspiration biopsy of thyroid nodules in children and adolescents. J Korean Med Sci.

