# Supplementary material for: A predictive model of thyroid malignancy using clinical, biochemical and sonographic parameters for patients in a multi-center setting
Source: BMC Endocr Disord. 2018 Mar 7;18:17. doi: 10.1186/s12902-018-0241-7 (PMC5842594; doi:10.1186/s12902-018-0241-7)
Supplement: Supplementary file 1 — Table S1. Clinical, biochemical and sonographic parameters of thyroid nodules ≤1 cm. (DOCX 17 kb) [file 12902_2018_241_MOESM1_ESM.docx]

Table S1

Clinical, biochemical and sonographic parameters of thyroid nodules ≤1cm

|  | Benign (n=157) | Malignant (n=104) | P value |
| --- | --- | --- | --- |
| Gender |  |  |  |
| Male, % | 14.4% | 16.8% | 0.535 |
| Age, y, mean (SD) | 47.8 (9.9) | 44.7 (9.3) | <0.001 |
| TGAb, % | 15.2% | 28.9% | <0.001 |
| Hypoechogenicity | 55.9% | 89.4% | <0.001 |
| Microcalcification | 19.9% | 42.3% | <0.001 |
| Central flow | 48.4% | 59.7% | 0.006 |
