# Supplementary material for: A predictive model of thyroid malignancy using clinical, biochemical and sonographic parameters for patients in a multi-center setting
Source: BMC Endocr Disord. 2018 Mar 7;18:17. doi: 10.1186/s12902-018-0241-7 (PMC5842594; doi:10.1186/s12902-018-0241-7)
Supplement: Supplementary file 2 — Table S2. Distribution of thyroid malignancy in patients with and without lymphocytic thyroiditis. (DOCX 15 kb) [file 12902_2018_241_MOESM2_ESM.docx]

Table S2

Distribution of thyroid malignancy in patients with and without lymphocytic thyroiditis

| Malignancy | Lymphocytic thyroiditis (n=208) | Non-lymphocytic thyroiditis  (n=2776) | P value |
| --- | --- | --- | --- |
| Presence | 58 | 418 | <0.05 |
| Absence | 150 | 2358 |  |
